# Identifying protective and risk factors for injurious falls in patients hospitalized for acute care: a retrospective case-control study

**DOI:** 10.1186/s12877-017-0627-9

**Published:** 2017-11-07

**Authors:** Emmanuel Aryee, Spencer L. James, Guenola M. Hunt, Hilary F. Ryder

**Affiliations:** 1 Geisel School of Medicine at Dartmouth, Hanover, NH USA; 2 Denver Health, 2900 Downing Street #404, Denver, CO 80204 USA; 3 Walter Reed National Military Medical Center, 10314 Strathmore Hall St #211, Bethesda, MD 20852 USA; 4 Dartmouth-Hitchcock Medical Center, Lebanon, NH USA; 5 Dartmouth-Hitchcock Medical Center, One Medical Center Drive, Lebanon, 03756 NH USA

**Keywords:** Accidental falls, Hospital, Injury, Risk factors

## Abstract

**Background:**

Admitted patients who fall and injure themselves during an acute hospitalization incur increased costs, morbidity, and mortality, but little research has been conducted on identifying inpatients at high risk to injure themselves in a fall. Falls risk assessment tools have been unsuccessful due to their low positive predictive value when applied broadly to entire hospital populations. We aimed to identify variables associated with the risk of or protection against injurious fall in the inpatient setting. We also aimed to test the variables in the ABCs mnemonic (**A**ge > 85, **B**ones-orthopedic conditions, anti-**C**oagulation and recent **s**urgery) for correlation with injurious fall.

**Methods:**

We performed a retrospective case-control study at an academic tertiary care center comparing admitted patients with injurious fall to admitted patients without fall. We collected data on the demographics, medical and fall history, outcomes, and discharge disposition of injured fallers and control patients. We performed multivariate analysis of potential risk factors for injurious fall with logistic regression to calculate adjusted odds ratios.

**Results:**

We identified 117 injured fallers and 320 controls. There were no differences in age, anti-coagulation use or fragility fractures between cases and controls. In multivariate analysis, recent surgery (OR 0.46, *p* = 0.003) was protective; joint replacement (OR 5.58, *P* = 0.002), psychotropic agents (OR 2.23, *p* = 0.001), the male sex (OR 2.08, *p* = 0.003) and history of fall (OR 2.08, *p* = 0.02) were significantly associated with injurious fall.

**Conclusion:**

In this study, the variables in the ABCs parameters were among the variables not useful for identifying inpatients at risk of injuring themselves in a fall, while other non-ABCs variables demonstrated a significant association with injurious fall. Recent surgery was a protective factor, and practices around the care of surgical patients could be extrapolated to reduce the in-hospital fall rates.

## Background

Inpatient falls are the most commonly reported adverse hospital event [1] and are associated with an increase in length of stay and a higher rate of patients being discharged to long-term care facilities. Inpatients who suffer an injurious fall incur significant costs and have increased morbidity compared with patients who do not fall [2].

Falls prevention and injury reduction is an active topic of research, with focus on preventative interventions and on risk factor identification or assessment. Randomized-controlled trials implemented to test interventions on fall prevention and injury reduction in many health care settings have been inconsistently successful [3]. A Cochrane review published on fall prevention in hospitals found that, of the interventions explored, only patient education in patients who were cognitively intact was statistically significant and consistently supported in the data, [3] and a later multi-center trial also demonstrated this [4]. In long-term care facilities and subacute wards, Vitamin D supplementation and interventions targeting specific risk factors on a case-by-case basis were consistently effective [3]. Other interventions investigated, including staff training, exercise programs, medication review and reorganization of care are inconsistently effective [3]. The differences between each study could suggest that hospitals have different strengths and weaknesses in the care provided to patients, and that when the data is pooled, the population differences specific to each study may be masked. In this context, risk factor identification is useful so that hospitals can review their own practices and change standard operating procedure to tailor to their patient population’s needs.

Many researchers have hoped to develop a useful risk assessment tool to identify patients who are at risk for injurious falls. The well-renowned and influential Institute of Health Improvement in the United States has recommended the use of the ABCs (Age > 85, Bones-orthopedic conditions, anti-Coagulation and recent surgery) to identify patients at risk to injure themselves in a fall; to date, there is no evidence to support this practice [5]. Other studies in the past have not supported the use of another risk-assessment, the STRATIFY tool, due to its low predictive value [6]. There is some new hope that a combination of the STRATIFY and FRAX bone health assessment could hold some utility and identifies injurious fallers more reliably [7], but it is still unknown if the positive predictive value is high enough to be cost-effective. Without the right clinical context, these risk assessment tools are overly sensitive and subsequently can result in time-intensive, costly, and often unsustainable interventions without real reductions in falls [6]. High-effort and low-yield interventions can lead to nursing burn-out as nurses perceive their efforts to prevent injuries do not make a difference.

Previous research has clearly elucidated patient risk factors for falling [8, 9] and the features of a hospital or a fall that lead to injurious falls [10, 11], but little research has been conducted on identifying patient risk factors for injurious fall during an acute hospitalization. Prior studies attempting to identify features of these people who are injured in a fall have suffered from insufficient sample size, [12] insufficiently robust control groups, [8, 13, 14] use of data of uncertain quality collected from adverse event tracking systems without corroboration from the medical record, [13–15] and a lack of consensus in defining injurious falls [16]. The NICE guidelines collected robust epidemiological data on risk factors for falls, but used data from long-term care facilities and the community [17]. A Cochrane review similarly focused on long-term care facilities [3]. Acute care hospital falls are different in several key ways, in that patients are in a new and unfamiliar environment, have an acute change in their functional status and are especially prone to delirium. It is difficult to extrapolate the research done in other settings to the unique experience of a hospitalization; but risk factor identification for in-hospital injurious falls is paramount to future investigations and intervention design so that best-care practices can be designed. One of the biggest successes in the field of in-hospital falls was a study that identified delirium as an independent risk factor, and as a result, the Hospital Elders Life Program (HELP) was formed to develop better and safer ways to care for delirious patients [18]. After its implementation, falls were reduced by two-thirds and the practices used were then implemented nationally in the United States, demonstrating the utility of evidence-based intervention design [18]. If we understand which patients are likely to injure themselves in a fall and which ones are not while admitted to the hospital, we will be able to more appropriately target hospital fall prevention resources.

## Methods

Our goal was to identify predictors of injurious falls in acutely hospitalized adults by conducting a retrospective case-control study. To study the predictors of injurious fall, we compared hospitalized patients who had a fall resulting in injury (cases) to hospitalized patients who did not have an injurious fall (control) in a retrospective case-control study. We chose to utilize a case-control study design as serious injurious falls are rare, but the exposures leading to the outcome are common.

### Study setting

The study occurred at an academic, tertiary-care center in northern New England with an average yearly admission of 20,500 patients. The facility’s 50 critical care beds and 346 inpatient acute care beds and provide medical, surgical, neurologic, pediatric, psychiatric, obstetric, oncologic and cardiovascular clinical services. The research protocol was reviewed and approved by the Dartmouth College Institutional Review Board, the Committee for the Protection of Human Subjects.

### Patient selection

#### Injured fallers

All adults admitted to the medical center between April 2011 and April 2014 were eligible for the study. Injurious falls (cases) were identified through our adverse event tracking system. The fall was verified in the patient’s medical record and data was collected from the medical record. Inclusion criterion for our cases was injurious fall in the inpatient setting, meaning any fall that resulted in an injury, from minor to serious; we included all injured inpatient fallers meeting inclusion criteria. We excluded pediatric patients and patients admitted to the psychiatric and obstetric wards from being cases or controls as falls in these populations could not be generalized to the general hospital population.

#### Control group

Three unique controls were identified for each case with selection criteria for factors not thought to be associated with fall – date of admission and length of stay prior to fall. These factors were chosen to neutralize the effect of seasons, changes in fall prevention strategy and increased length of stay seen with inpatients who fall. Controls were identified through the medical center’s data warehouse. In three instances, cases had been erroneously identified as controls and were eliminated from the control group. Twenty-eight other identified controls met exclusion criteria and were removed. Presence or absence of a fall in the control group were not selection criteria for the control group, however, there were no falls in the control group.

### Variables

We collected data on demographics, medical and social history, medication and fall history, mobility, and outcomes of injured fallers and control patients, as well as on the injurious fall, using standardized operational definitions of variables found to be predictive of falls and fall-related injury in the literature.

### Sample size calculation

The number of inpatient injured fallers is fixed. To calculate sample size, we determined the baseline prevalence of osteoporosis, cognitive impairment, female patients and patients older than 85 in the hospital using published data from the Healthcare Cost and Utilization Project [19]. We assumed a two-sided α-level of 0.05, and statistical power of 80% and found that with predicted enrollment, our study was powered to detect what was thought to be a clinically significant 10–15% difference in cases and controls among the outcome variables of interest. Power calculations for logistic regression with binary covariates were performed using algorithms described by Demidenko [20]. An attempt was made to calculate the minimal detectible difference, however standard deviations or variance for baseline prevalence for the categorical variables of interest could not be determined because of insufficient published data. Based on these calculations, we aimed to collect data on a minimum of 100 injured fallers and 300 control patients.

### Data collection

All inpatient falls are entered, per hospital protocol, into an electronic adverse event tracking system. Cases were identified by querying this system for falls and screening for injurious falls. Controls were identified by experts within the data warehouse using our pre-specified criteria. A trained research assistant (EA) reviewed and abstracted information through a retrospective chart review of the medical record into an electronic database; all patient data used in this study was de-identified. To ensure high-quality data abstraction, the primary investigator (HFR) randomly abstracted 25 charts and a kappa of 0.86 indicated high agreement.

### Statistical analysis

We performed a sex-specific analysis of age as a continuous variable, using ROC analysis, to determine whether an age-related threshold of risk might exist for women and for men, but found no such threshold. We used univariate logistic regression to calculate unadjusted odds ratios with 95% confidence intervals for each of the study variables for injurious fall. A multivariate logistic regression was performed on variables found to vary meaningfully between injured fallers and controls in univariate logistic regression (based on *p*-values less than 0.10 in univariate analysis). We calculated the impact of injurious fall on outcomes including discharge disposition and length of stay. Data analysis was conducted with Stata version 13 and was considered statistically significant if *p* < 0.05 (two-tailed α).

## Results

### Study population

We identified 117 injured fallers and 320 controls. There were no significant differences between the two groups in age or body mass index (BMI) (see Table 1). A smaller proportion of the cases were female compared to controls (0.32 compared to 0.48, *p* = 0.002). There were no significant differences in admission source. While we did not select for or against non-injurious falls in the control group, no control patients experienced a fall (with or without injury) during their hospitalization. The prevalence of orthopedic conditions was similar in both populations.

**Table 1:** Demographics and outcomes of injured fallers and matched controls*

Variable	Patients with injurious falls (*n* = 117) [number (%)]	Controls (*n* = 320) [number (%)]	*P* value
Demographics			
Mean age (SD)	65.09	63.15	0.2783
Male sex	80 (68.4)	166 (54.9)	0.0020
Average BMI (SD)	27.39	28.14	0.4261
Admission source			
Missing	2 (1.7)	1 (0.30)	0.453
Home	80 (68.4)	218 (68.1)	
Hospital transfer	33 (28.2)	96 (30.0)	
Rehab facility	1 (0.85)	0 (0)	
SNF	3 (2.6)	5 (1.6)	
	117	320	
Discharge disposition			
Missing	0 (0)	1 (0.3)	0.119
Deceased	8 (6.8)	15 (4.7)	
Home	58 (49.6)	188 (58.8)	
Home Hospice	1 (0.9)	0 (0)	
Hospital transfer	10 (8.5)	11 (3.4)	
Rehab Facility	17 (14.5)	46 (14.4)	
SNF	23 (19.7)	59 (18.4)	
Length of stay	16.40171	14.92812	0.3113

* Footnote: Three unique controls were matched to each case. Controls were matched on admission date of the case (within seven days of admission) and length of stay (length of stay ≥ pre-fall LOS of case)

### Fall circumstances

The majority of injurious falls (80.3%) occurred on a hospital ward (see Table 2). Three-quarters of injurious falls occurred on medical units and one-quarter on surgical units. Falls were evenly distributed between day and night shifts. Only a small proportion of patients (22.2%) suffered a witnessed fall; 59% were found on the floor by hospital staff. In 43.6% of cases the fall was related to toileting. Seventeen patients (14.5%) fell while on bed rest; other patients had activity orders allowing some degree of ambulation. Fifteen patients (12.8%) were noted to be confused at the time of the fall.

**Table 2:** Circumstances of injurious fall

Characteristic	Number of patients (%)
Location	
Intensive Care Unit	11 (9.4)
Step-Down Unit	12 (10.3)
Ward Unit	94 (80.3)
Medical Unit	90 (76.9)
Surgical Unit	27 (23.1)
Time	
Day shift (7:00 AM to 6:59 PM)	61 (52.1)
Night shift (7:00 PM to 6:59 AM)	56 (47.9)
Character of fall	
Witnessed fall	26 (22.2)
Self-reported fall	19 (16.2)
Found on floor	69 (59.0)
Unknown	3 (2.6)
Assist type	
Unassisted fall	106 (90.6)
Assisted fall	8 (6.8)
Unknown	3 (2.6)
Fall mechanism	
Slip/trip	86 (73.5)
Dizzy/syncope	10 (8.5)
Weakness	3 (2.6)
Unknown	18 (15.4)
Fall-related activity	
Need for elimination	51 (43.6)
No need for elimination	51 (43.6)
Seizure	1 (0.8)
Unknown	14 (12.0)
Assigned activity level	
Bedrest	17 (14.5)
Ambulatory	100 (85.5)
Mental status	
Alert	101 (86.3)
Confused	15 (12.8)
Unknown	2 (1.7)

### Type and severity of injury

To categorize and define types of falls, we adopted widely used standardized definitions [16, 21] A fall was defined as an event in which a patient unintentionally came to rest on the floor, ground or lower level. A minor injurious fall was defined as a fall that resulted in minor bruising, sprains, cuts abrasions or a reduction in physical function for at least three days or requiring minor medical intervention; excluding serious injurious fall. A moderate injurious fall was a fall that resulted in wounds, bruises, sprains, cuts requiring a medical/health professional examination such as physical examination, x-ray, or suture. A serious injurious fall was defined as a fall resulting in medically recorded fracture, head or internal injury requiring accident and emergency or inpatient treatment, including hematoma requiring monitoring or loss of consciousness, subdural hematoma or other moderate or major head trauma, cardiac arrest or death. The majority (83.8%) of injurious falls resulted in minor injuries (see Table 3). Of the remainder, 13.7% sustained a moderate injurious fall, and 2.6% sustained a serious injurious fall (Table 3). Of the three serious injurious falls, two major injuries and one death occurred. Lacerations, skin tears, and contusions, abrasions, or bruises were the most common types of injury (Table 3). The face was the most common site of injury; arm, leg, and head were also common (Table 3).

**Table 3:** Injury severity and type

Characteristic	Number of patients (%)
Injury severity level	
Minor	98 (83.8)
Moderate	16 (13.7)
Serious	3 (2.6)
Type of injury	
Laceration	15 (12.8)
Contusion, abrasion, or bruise	29 (24.8)
Bleeding	11 (9.4)
Skin tear	19 (16.2)
Pain or soreness	9 (7.7)
Bump	6 (5.1)
Other	8 (6.8)
Not recorded	22 (18.8)
Location of injury	
Head (not including face)	14 (12.0)
Arm	14 (12.0)
Leg	14 (12.0)
Face	31 (26.5)
Foot/ankle	6 (5.1)
Trunk/back	8 (6.8)
Hand/wrist	4 (3.4)
Other	4 (3.4)
Not recorded	25 (21.3)

Due to multiple types and locations of injury, these numbers exceed the number of patients

### Results of univariate analysis

We compared the demographics, admission source, medical history, active treatments and fall characteristics of injured fallers and control patients (Table 4). There were significantly more men in the injured fallers group (OR 2.0, CI (1.28–3.13), *p* = 0.002). There were no differences in history of cognitive impairment, fragility fracture or active smoking between the two groups. Recent surgery was protective (OR 0.45, *p* = 0.001). Injured fallers had a significantly higher mean Charlson Comorbidity Index (21) (6 vs 5, *p* = 0.001). There was a significant association between use of psychotropic agents (OR 2.54, *p* < 0.0001), use of vasoactive agents (OR 1.75, *p* = 0.011), history of joint replacement (OR 3.73, *p* = 0.011) and being a faller with a minor, moderate or serious injury. There was an association between being assessed at risk to fall and an injurious fall, but it was not significant. However, history of a fall (OR 2.69, *p* = 0.001) was significantly associated with being an injured faller.

**Table 4:** Univariate analysis of predictors of injurious fall

	Variable	Patients with injurious falls (*n* = 117) [number (%)]	Controls (*n* = 320) [number (%)]	OR	CI	*P* Value
Demographics	Age > 70	48 (41)	111 (34.7)	1.31	(0.85–2.02)	0.223
	Male sex	80 (68.4)	166 (54.9)	2	(1.28–3.13)	0.002
Medical history						
	Cognitive Impairment	20 (17.1)	33 (10.3)	1.79	(0.98–3.27)	0.057
	History of fragility fracture	12 (10.3)	30 (6.3)	1.71	(0.81–3.63)	0.159
	History of joint replacement	9 (7.7)	7 (2.2)	3.73	(1.36–10.25)	0.011
	Recent surgery	32 (27.4)	146 (45.8)	0.45	(0.28–0.71)	0.001
	Current smoker	20 (17.9)	45 (14.9)	1.25	(0.70–2.22)	0.455
	Mean Charlston Comorbidity Index (SD)	6 (SD 3.6)	5.0 (SD 2.7)			0.001
Active treatments						
	CNS agents	79 (67.5)	144 (45.0)	2.54	(1.63–3.97)	<0.0001
	Vasoactive agents	71 (60.7)	150 (46.9)	1.75	(1.14–2.69)	0.011
	Therapeutic dose anticoagulants	19 (16.2)	47 (14.7)	1.13	(0.63–2.01)	0.688
Characteristics						
	Assessed “at risk to fall”	57 (48.7)	123 (38.6)	1.51	(0.99–2.32)	0.057
	History of fall	27 (23.1)	32 (10.0)	2.69	(1.53–4.73)	0.001

### Results of multivariate analysis

In multivariate logistic regression analysis, recent surgery (OR 0.46, *p* = 0.003) was significantly protective (see Table 5). Male sex (OR 2.08, *p* = 0.003), history of joint replacement (OR 5.58, *p* = 0.002), use of psychotropic agents (OR 2.23, *p* = 0.001), and prior history of a fall (OR 2.08, *p* = 0.02) were significantly associated with being an injured faller. Using this multivariate logistic model yielded 75.69% accuracy (sensitivity 26.50%, specificity 93.73%, AUC 0.742) in correctly classifying fall versus not-fall patients during in-sample validation. Testing this same multivariate model including all ABCs variables (age, joint replacement, fracture, anti-coagulation, recent surgery) yielded 76.89% accuracy (sensitivity 30.77%, specificity 93.73%, AUC 0.751), and using the ABCs alone yielded a 73.17% accuracy (sensitivity 9.40%, specificity 96.55%, AUC 0.649). Significantly more cases than control patients used psychotropic agents during their hospitalization. Psychotropic drugs used most frequently in both populations were primarily benzodiazepines, anti-epileptics, sleep aids and anti-depressants; but included anti-psychotics, sleep aids, CNS stimulants, narcotics, and dopaminergic antiparkinsonism agents. A significantly higher proportion of injured fallers used two or more agents (41.8% vs 13.8% *p* < 0.0001) and two or more classes of psychotropic agents than control patients (32.5% vs 12.5%, *p* < 0.0001).

**Table 5:** Multivariate analysis of predictors of injurious fall

Variable	OR	CI	*P* Value
Male sex	2.08	(1.28–3.45)	0.003
History of joint replacement	5.58	(1.84–16.9)	0.002
Recent surgery	0.46	(0.28–0.76)	0.003
Charlston Comorbidity Index	1.08	(1.00–1.17)	0.06
Use of CNS agents	2.23	(1.39–3.60)	0.001
Use of vasoactive agents	1.6	(1.00–2.58)	0.051
Cognitive Impairment	1.13	(0.59–2.17)	0.715
Assessed “at risk to fall”	1.37	(0.86–2.20)	0.19
History of fall	2.08	(1.12–3.85)	0.02

### Post-fall outcomes

Collectively, injured fallers had a slightly longer length of stay compared to their matched controls but this difference was not statistically significant (See Table 6). There were no significant differences in the discharge dispositions of injured fallers and control patients.

**Table 6:** Outcomes of injurious fallers and controls

Variable	Patients with injurious falls (*n* = 117) [number (%)]	Controls (*n* = 320) [number (%)]	*P* value (Fischer’s exact test)
Discharge disposition			
Missing	0 (0)	1 (0.3)	0.119
Deceased	8 (6.8)	15 (4.7)	
Home	58 (49.6)	188 (58.8)	
Home Hospice	1 (0.9)	0 (0)	
Hospital transfer	10 (8.5)	11 (3.4)	
Rehab Facility	17 (14.5)	46 (14.4)	
SNF	23 (19.7)	59 (18.4)	
Length of stay	16.40171	14.92812	0.3113

## Discussion

Injurious inpatient falls are all too frequent events that lead to significant morbidity and mortality and increased healthcare costs. Because of this, there has been significant attention given to identifying patients likely to fall or to injure themselves in a fall as a fall prevention strategy. This retrospective case-control study suggests that the ABCs alone as a risk-assessment tool lack the predictive power necessary to identify inpatients at moderate or severe risk of injuring themselves in a fall, despite its recommended use by the Institutes of Health Improvements and adoption widely within the United States. While risk assessments for fall prevention are controversial [22], other clinical risk factors may be more successful in predicting injurious fall and developing more targeted interventions.

The male sex, history of a fall, history of a joint replacement, and use of psychotropic agents were significant risk factors in multivariate analysis. The correlation between being male and an increased risk of falls has recently been described by Toyabe et al. [7]. History of falling is known to predict future falls in the inpatient setting [23, 24]. Our study demonstrates that those who have fallen before are at high risk of injury when they fall again. A fall history should be documented upon admission to the hospital and fallers should be offered special precautions to prevent future injuries. History of joint replacement is a known risk factor for persistent gait disturbance [25, 26] and gait disturbance is a known risk factor for inpatient falls [27]. In our study, joint replacement could be a proxy for persistent gait disturbance.

Cognitive impairment, defined as a documented history of delirium, dementia or other cognitive impairment, was not a significant risk factor in our analysis, although it has been described in other studies [18]. It is possible that investigation of this important limitation on cognition in our study was limited by underreporting of this information, as chart review was the primary modality of data collection. It is also possible that recent adoption of better care practices for cognitively impaired patients, such as the widespread understanding and implementation of practices promoted by the HELP study, has led to a decrease in the importance of this factor as a risk factor. Use of psychotropic agents, including use of multiple agents and multiple classes of drugs, was significantly associated with injury. These drugs have significant movement-related side effects, including ataxia and bradykinesia, which could lead to imbalance, inability to compensate, and a fall resulting in injury. Interestingly, not only was there more use of these agents by injured fallers, the polypharmacy of such agents was significant. Combining multiple drugs and multiple classes of drugs is known in many instances to increase bioavailability of drug [28] and lead to a synergistic potentiation of side effects [29, 30] predisposing polypharmacy users to injurious fall. Our study adds further evidence to the argument that psychotropic agents should be used with caution. Further, the strength of this association argues that use of psychotropic agents, and especially multiple agents and multiple classes of agents, should be considered a risk factor for injurious fall.

In the present study, history of recent surgery was a significant protective factor. This has not been described previously. In collecting a larger sample size and selecting a new population of controls (no falls versus non-injurious falls), our study was better able to avoid type II error and uncover this correlation. The protective effect of recent surgery may be due to differences in how a surgical patient’s toileting is managed. Toileting is the most common activity linked to injurious falling in our study, implicated in 43.6% of documented falls, and is known to lead to falls with injury in other studies as well [31]. Patients who have recently undergone surgery are more likely to toilet while in bed, either using bed pan or urinary catheter [7]. Perhaps this finding can offer guidance on future fall prevention strategies. If in-bed toileting is indeed the reason for less falls after a surgery, we can optimize toileting practices for at-risk patients, such as those with joint replacements, history of falls or receiving a risky medication.

The injured fallers in our study were like those found in hospitals across the country. The demographics of injured fallers, circumstances of injurious fall, severity of injury sustained after inpatient fall, and types of injuries sustained after inpatient falls were similar to those found in other studies [12, 14, 15, 31, 32]. Our findings differ from other studies of injured fallers because our *control* group varied significantly from those of other studies. Other published studies [12, 14, 15, 31, 32] use non-injured fallers as a control group. While this group is easier to identify than finding matched inpatient non-fallers, to truly identify patients at risk to injure themselves in a fall, the control group must represent *all* those at risk to injure themselves in a fall – all inpatients – not patients who *do not* injure themselves in a fall. We believe the differences in our findings results from our choice of control group. Because of this, our study had more sensitivity to find statistically significant risk factors than other case-control studies conducted previously.

Our study has several limitations. First, our study was performed at a single academic medical center in the United States and included acutely ill patients from both medical and surgical inpatient units. Our conclusions may not be generalizable to other hospitals with different patient demographics. Fall prevention strategies differ from institution to institution and country to country. Risk factors for injurious fall may also differ according to fall prevention strategies in place, limiting the generalizability of our findings, though as described in methods above we included all possible risk factors for falls that were available in our data in the conducted analysis. Studies at other institutions, using matched non-falling controls, should be performed to validate our work. Second, we relied on the adverse event tracking system to identify cases and therefore may have missed some injurious falls. However, prior studies have demonstrated that falls, especially falls with injury, are accurately reported through such hospital-mandated systems, and in analyzing injurious falls, and omitting non-injurious falls, we maximized the opportunity that our system appropriately captured events. Third, while we attempted to identify controls with characteristics not associated with falls (date of admission and length of stay prior to fall), such a process can select controls with similar characteristics to cases, potentially over-representing high risk patients within the control group. Additionally, while we collected data on variable found to be predictive of falls and fall-related injury in the literature, we cannot rule out the potential that these risk and protective factors are confounders related to an as yet-to-be-discovered underlying factor. Finally, while our enrollment was larger than most published studies of injured fallers, our small sample size may have put us at risk of type II errors. More research is needed to generalize our results and further clarify risk and protective factors for injurious fall.

## Conclusion

Understanding risk factors that predispose patients to injure themselves in a fall in the hospital is crucial to implementing evidence-based and resource-efficient interventions aimed at preventing such events. Age and anti-coagulation, while theoretically appealing as risk factors for an injurious fall, were similar in injurious fallers and controls, and so were not useful in identifying in-hospital fallers. Our study did find statistically more frequent falls in patients who are male, have a history of falls, have had joint replacements, and were given psychotropic drugs. Conversely, recent surgery was protective in this study, which was a novel finding, and could suggest a role for more in-bed toileting for patients with a history of falls, or other risk factors.

The key to preventing injuries from falls is not merely to identify those individuals at risk for falling, but to identify those at risk for injury. Research focused on identifying patients at high-risk to fall has not prevented injuries from falls [33–35]. If equal attention is given to identifying patients at high-risk of injurious falls and creating tools to assess risk of injurious falls, we might reduce hospital fall injuries by targeting this smaller group of patients. This study provides the first evidence-based, patient-specific factors to identify those at risk for injury from falling while hospitalized.

## Abbreviations

ABCs: Age > 85, Bones-orthopedic conditions, anti-Coagulation and recent surgery
BMI: Body mass index
OR: Odds ratio
